# Does financial inclusion and information communication technology affect environmental degradation in oil-producing countries?

**DOI:** 10.1371/journal.pone.0298545

**Published:** 2024-03-20

**Authors:** Isbat Alam, Lu Shichang, Saqib Muneer, Khalid Mahsan Alshammary, Muhammad Zia ur Rehman

**Affiliations:** 1 College of Business Administration, Liaoning Technical University, Fuxin, China; 2 Department of Economics and Finance, University of Ha’il, Ha’il, Saudi Arabia; 3 School of Distance Education, Universiti Sains Malaysia, Gelugor, Malaysia; 4 Faisalabad Business School, National Textile University, Faisalabad, Pakistan; Islamia University of Bahawalpur, PAKISTAN

## Abstract

Advances in financial inclusions have contributed to economic growth and poverty alleviation, addressing environmental implications and implementing measures to mitigate climate change. Financial inclusions force advanced countries to progress their policies in a manner that does not hinder developing countries’ current and future development. Consequently, this research examined the asymmetric effects of information and communication technology (ICT), financial inclusion, consumption of primary energy, employment to population ratio, and human development index on CO_2_ emissions in oil-producing countries (UAE, Nigeria, Russia, Saudi Arabia, Norway, Kazakhstan, Kuwait, Iraq, USA, and Canada). The study utilizes annual panel data spanning from 1990 to 2021. In addition, this study investigates the validity of the Environmental Kuznets Curve (EKC) trend on the entire sample, taking into account the effects of energy consumption and population to investigate the impact of financial inclusion on environmental degradation. The study used quantile regression, FMOLS, and FE-OLS techniques. Preliminary outcomes revealed that the data did not follow a normal distribution, emphasizing the need to use quantile regression (QR). This technique can effectively detect outliers, data non-normality, and structural changes. The outcomes from the quantile regression analysis indicate that ICT consistently reduces CO_2_ emissions in all quantiles (ranging from the 1st to the 9th quantile). In the same way, financial inclusion, and employment to population ratio constrains CO_2_ emissions across each quantile. On the other side, primary energy consumption and Human development index were found to increase CO_2_ emissions in each quantile (1st to 9th). The findings of this research have implications for both the academic and policy domains. By unraveling the intricate interplay between financial inclusion, ICT, and environmental degradation in oil-producing nations, the study contributes to a nuanced understanding of sustainable development challenges. Ultimately, the research aims to guide the formulation of targeted policies that leverage financial inclusion and technology to foster environmentally responsible economic growth in oil-dependent economies.

## 1. Introduction

Global economies dependent on natural resources have witnessed remarkable economic growth and development in recent decades. Redundant and Exploitation of natural resources and their scarcity have played an important role in driving economic and financial advancement [1, 2]. However, rapid economic growth has frequently come at the expense of environmental quality. To address this issue, countries have been forced to implement policies promoting economic growth and environmental sustainability, known as green growth [3, 4]. The Climate Change Conference in Glasgow (COP26) emphasized coal consumption power reduction and the phase-out of wasteful fossil fuel subsidies as critical measures toward a low-carbon future. Kuznets [5] Presented the concept of an inverted U-shaped link between economic expansion and environmental quality, first postulated by [5]. The Environmental Kuznets Curve (EKC) suggests that when an economy progresses and reaches specific levels of income per capita, the adverse environmental consequences of economic growth can be mitigated, leading to improved environmental conditions. This association means that degradation of the environment is more likely during the early stages of economic expansion, but if certain income thresholds are met, additional growth is associated with improved environmental quality [6, 7]. Whether economic growth is viewed as a threat or an opportunity for the environment, the financial sector is critical in promoting economic expansion and affecting environmental results [8]. As hypothesized, increased economic activity, growth, and demand for financial interconnection encourage the financial sector to broaden its range of services and goods, as financial support is viewed as a stimulant for continuous and sustainable growth. Financial inclusion has emerged as a viable tool for minimizing the degradation of the environment while providing inexpensive, long-term, and dependable energy services. Financial inclusion has been widely debated and recognised for its role in encouraging economic growth as an essential component of long-term financial and economic development [9, 10]. Financial inclusion policies act as proxies for various development initiatives and are closely associated with the 2030 sustainable development goals (SDGs). Financial inclusion also supports capital allocation, circulation, pooling, and savings by assuring the inclusion of varied segments of society in the financial system, hence addressing the financial needs of individuals [11]. Advances in sophisticated technology instruments have increased energy production and consumption in the globalization period, creating problems and concerns relating to environmental issues [12]. Financial inclusion, in theory, may negatively impact the environment by improving access to financial products and services, which can lead to increased output and industrial growth, resulting in the degradation of the environment [13]. However, the financial sector has the potential to play a significant role in addressing environmental concerns, particularly those associated to energy emissions, by supporting the deployment of green technology that reduce the environmental impacts of non-renewable energy consumption [14]. Modern technological breakthroughs can promote green economic growth that creates a clean and green environment while preserving and maintaining the environment [15]. As a result, financial inclusions can be a critical component in lowering CO_2_ emissions [16]. [17], financial inclusion can help disadvantaged groups, Farmers, for example, who want funding to invest in more cost-effective and ecologically friendly equipment. The rise of the ICT era has resulted in several economic benefits [18]. ICT supports trade by opening up new international marketplace and providing opportunities for developing and developed economies. It also improves education, health, and manufacturing tools by facilitating information and communication. Undoubtedly, ICT is an essential to accomplishing the Sustainable future objectives [19]. ICT considerably promotes all elements of sustainable economic growth and has become critical for economic and corporate decision-making, resulting in cost savings and increased productivity [15]. ICT provides several options, with cost reductions and convenience being key aspects [9]. The study outcomes of [20] suggested that by incorporating renewable energy into many parts of green ICT, ICT can contribute to energy efficiency. By utilising artificial intelligence and grid optimisation technologies, ICT plays a vital role in reaching energy-saving targets [21]. However, the global expansion of ICT consumer gadgets such as e-government, mobile banking, and social networking considerably impacts energy consumption. The findings revealed that growing energy demand raises the amount of ICT-related greenhouse gas (GHG) emissions, finally leading to the degradation of the environment [18]. Despite of large body literature on many elements of environmental degradation, there is still a major vacuum in understanding the factors that contribute to the environmental degradation. A recent study has incorporated new aspects into analysing environmental degradation causes. In this light, the research gap identified in our paper is a lack of studies examining whether the ICT diffusion in ten oil-producing nations can attenuate the relationship between financial inclusion and environmental degradation. This study is motivated by the conviction that unravelling the complex web of interactions between financial inclusion, ICT, and environmental degradation is essential for informed policy formulation. By gaining insights into the specific challenges and opportunities faced by oil-producing countries, we can develop targeted strategies that align economic progress with environmental stewardship. In the face of escalating climate change concerns and the imperative for sustainable development, this research aspires to contribute valuable knowledge to academia, policymakers, and practitioners alike. The findings aim to guide the design of interventions that harness the positive aspects of financial inclusion and technology to foster environmentally responsible economic development in oil-producing countries, thereby striking a balance between economic prosperity and ecological well-being. This study intends to considerably contribute to the existing knowledge base. The study’s contribution can be divided into four major categories. First, the availability of natural resources in these countries has contributed to their rapid economic growth, which has resulted in rising environmental challenges. Second, because the sample comprises countries from various areas with differing degrees of financial technology, human capital, financial development, and ICT the impact of these variables on environmental quality are predicted to vary greatly. As a result, environmental challenges may be prioritised differently throughout different stages of development. Oil-producing countries, for example, may produce and utilize more energy while experiencing low financial inclusion development, and their ICT structure may lag behind the rest of the globe, resulting in limited gains from green ICT practises. Third, unlike previous studies [21], which concentrated on the impact of numerous environmental indicators, this study looks into the possible benefits of ICT and financial inclusion on environmental degradation. Finally, when contrasted to utilising the QR is used in this study to evaluate the influence of several indicators based on the response variable’s conditional median, allowing for more meaningful and robust estimations. The study’s objectives are twofold: (1) to explore the influence of economic growth, financial inclusion, and ICT spread on the degradation of the environment in the oil-producing countries, and [22] to see if ICT diffusion helps to attenuate the association between financial inclusions and environmental degradation.

The research is organized as follows: The subsequent section provides an overview of the existing literature and presents a summary of the study’s hypotheses. Section (3) introduction of data and materials used. Section (4) discusses and presents the study’s findings. Finally, Section (5) outlines the major policy implications of the work.

## 2. Literature review

### 2.1 Nexus between financial inclusion and environmental degradation

There is controversy surrounding the idea of financial inclusion because of its potential to help people experiencing poverty and those in need by fostering economic growth [23]. Two major theoretical perspectives regarding financial inclusion’s impact on environmental quality exist. From a theoretical standpoint, financial inclusion is seen as beneficial for various sectors of the economy as it provides funding for expansion and growth [24]. However, encouraging businesses to enhance production-driven economic activity growth causes a surge in energy consumption and, as a result, degradation of the environment [25]. Studies by [24, 26] claim that consumers may finance high-energy consumer products like vehicles, coolers, and air conditioners because financial services are inclusive, leading to increased energy consumption that is harmful to the environment [24], demonstrates the negative effects of financial development on Turkey’s environmental degradation. [27] The Eurozone’s environmental degradation is negatively impacted by financial inclusion, but the spread of innovation lessens this impact [28]. [29] Cite more evidence to support the argument that higher CO_2_ emissions brought on by financial development negatively influence environmental quality in eight emerging economies. However, there are arguments in favour of financial inclusion’s beneficial contribution to improving environmental quality. For environmental protection, funding initiatives that advance technological innovation in sustainable energy are essential [30]. It is beneficial for environmental protection and the fight against environmental degradation so that money is readily available to various social groups with low interest rates and reasonable financial needs to finance productive activities [31]. The recent empirical studies conducted by [24, 32] demonstrate that increased financial inclusion leads to improved environmental outcomes, thereby the positive impact of financial inclusion supporting on environmental deterioration. But, the study of [33] shows only in developed countries, consistent with the Environmental Kuznets Curve (EKC) theory.

There is limited empirical data on the moderating influence of ICT in reducing environmental risks associated with financial inclusion, even though the environmental impact of financial inclusion is still unknown [20]. [34] A contrastive assessment of how financial inclusion affected environmental quality considering ICT diffusion. They found that the interaction between ICT transmission and financial inclusion reduced emissions, particularly via mobile use. However, this moderating effect was not observed. When internet use was considered, Similar findings were supported by [35, 36]. In the case of developing countries, they argued that efficient money management within a sophisticated financial system results in technological progress that reduces environmental impact. In contrast, [37] discovered financial growth and rising ICT investment. In light of the contradictory results of the moderating effects of ICT use on the association concerning financial inclusions and environmental degradations, we propose the subsequent solutions.

### 2.2 Nexus between information and communication technology and environmental degradation

ICT is a complex and multifaceted phenomenon encompassing various components such as hardware, communication equipment, and software services. It has significantly transformed people’s lives worldwide [38]. The widespread use of ICT has helped remove hurdles in business contexts and facilitated the movement of products and services, resulting in wealth and economic growth. ICT claims to substantially impact environmental sustainability and quality, particularly by switching to more environmentally friendly technology [39]. According to [40], Investing wisely in ICT can reduce energy and resources essential for manufacturing, opening the door to energy protection and carbon reduction. By simplifying intelligent design and manufacturing tools, it helps to rapidly adopt the latest, economical, and energy-efficient technologies by reducing the need for energy-intensive equipment [40], ultimately contributing in the long run to a sustainable environment [41]. This means that innovations in the ICT sector can help reduce the negative environmental impact of financial innovation. It enables economically disadvantaged groups to access financial products at competitive rates by adopting technological improvements to reduce pollution emissions. ICT is advancing rapidly, and wireless ICT encourages the development of renewable energy sources and offers useful techniques to reduce emissions [42]. The findings support [43] the idea that environmental innovation can prevent environmental problems, especially in countries claiming that the benefits of ICT development are only realized when a certain level of income is achieved. EKC for ICT diffusion, which posits that the benefits of ICT development are only felt until a certain level of wealth is met, is also supported by these figures [27]. Proof from China also emphasizes the important part that innovation plays in preventing environmental degradation [20]. But although helping to replace energy-efficient, low-carbon devices, ICT proliferation can also threaten the environment due to the energy use associated with ICT infrastructure and equipment [44]. According to recent studies, ICT innovation may damage the environment by raising carbon emissions and increasing ICT use. Using wavelet tools [45], provide data showing that Japan’s emissions have increased due to ICT advancements. The positive effects of ICT on environmental quality only become noticeable when a certain degree of ICT development is attained, according to other research that has proven the presence of a non-linear association between ICT and CO_2_ emissions [46].

### 2.3 Nexus between economic development and environmental degradation

Over the past few decades, the majority of the work that has been written on the topic of the connection between economic growth and environmental quality has been based on the EKC hypothesis [47]. According to the EKC theory, the inverted U-shaped curve illustrates the association between economic growth and environmental degradation. Kuznets [5] who noted a connection between rising per capita income and income disparity, is credited with developing this hypothesis. Environmental economists such as [48] examined the potential relationship between environmental degradation and economic success using Kuznets’ theory. They suggested a framework that divided economic expansion into three phases: size, structural, and combined effects. Before the turning point, the scale effect takes place. However, the second stage applies to industrialized base economies. The last phases are related to industrialized base economies, although the scale effect is more important in rising economies that rely on non-renewable energy sources [31]. As a result, the EKC theory claims that environmental degradation is more pronounced in the initial phases of economic development. Afterwards, a certain level of affluence is reached, and the trend reverses, resulting in increased economic activity and improved environmental quality [3]. Numerous conclusions have been drawn from empirical research on the EKC hypothesis, including [49]. An ARDL study in Bangladesh found that economic expansion causes environmental degradation over the medium and long term; GDP is used as a metric for economic growth. The study also found a relationship between declining human capital and adverse environmental impacts [50]. The human index’s impact on environmental quality in South Asian countries was investigated. The panel modelling results revealed a positive relationship between the human index and environmental quality; nonetheless, they discovered a divergence from the EKC hypothesis, which implies a positive relationship between economic growth and climate degradation. Their findings are consistent with the findings of [47] covers a subset of African economies where the inverted U-shaped association between per capita income and environmental quality does not occur, providing no evidence for the EKC hypothesis’s validity [51]. Using a long-run ARDL Co-integration approach, the researchers discovered that human capital reduces CO_2_ emissions in Pakistan.

### 2.4 Gaps in the literature

Numerous research studies have been conducted across various areas, economic systems, and political alignments to investigate the association between financial inclusion and environmental quality [52]. However, as far as we know, no study has particularly explored this relationship in the context of oil-producing countries. Since oil-producing countries have different financial and economic systems and technical advances, it is interesting to explore this link to acquire more useful insights for these countries. Previous research concentrated on the role of financial inclusion and information and communication technologies in reducing environmental degradation [53]. However, due to limitations in their unit of analysis, the results of these investigations did not yield definitive findings. As a result, re-examining the impact of ICT and financial inclusion on environmental degradation in selected countries, including oil-exporting nations like the United States, Canada, and Norway, as well as emerging economies like Saudi Arabia, Russia, Kuwait, and the United Arab Emirates, could significantly contribute to the existing literature. Furthermore, researching the role of ICT and financial inclusion in major oil producing countries might reveal significant implications for these countries and inform policies fostering long-term growth in the energy industry. Understanding how increasing ICT spread may contribute to environmental preservation if a negative Co-efficient with financial inclusion is discovered may be beneficial in this regard.

## 3. Methodology

This part of the research explains model construction and clarifies the theoretical foundation. Section 3 reviews the study’s data and the key econometric procedures used to investigate the association between factors such as the human development index and its squared term, population, energy consumption, ICT diffusion, financial inclusion, and environmental degradation.

### 3.1 Theoretical framework and model construction

**Fig 1:**
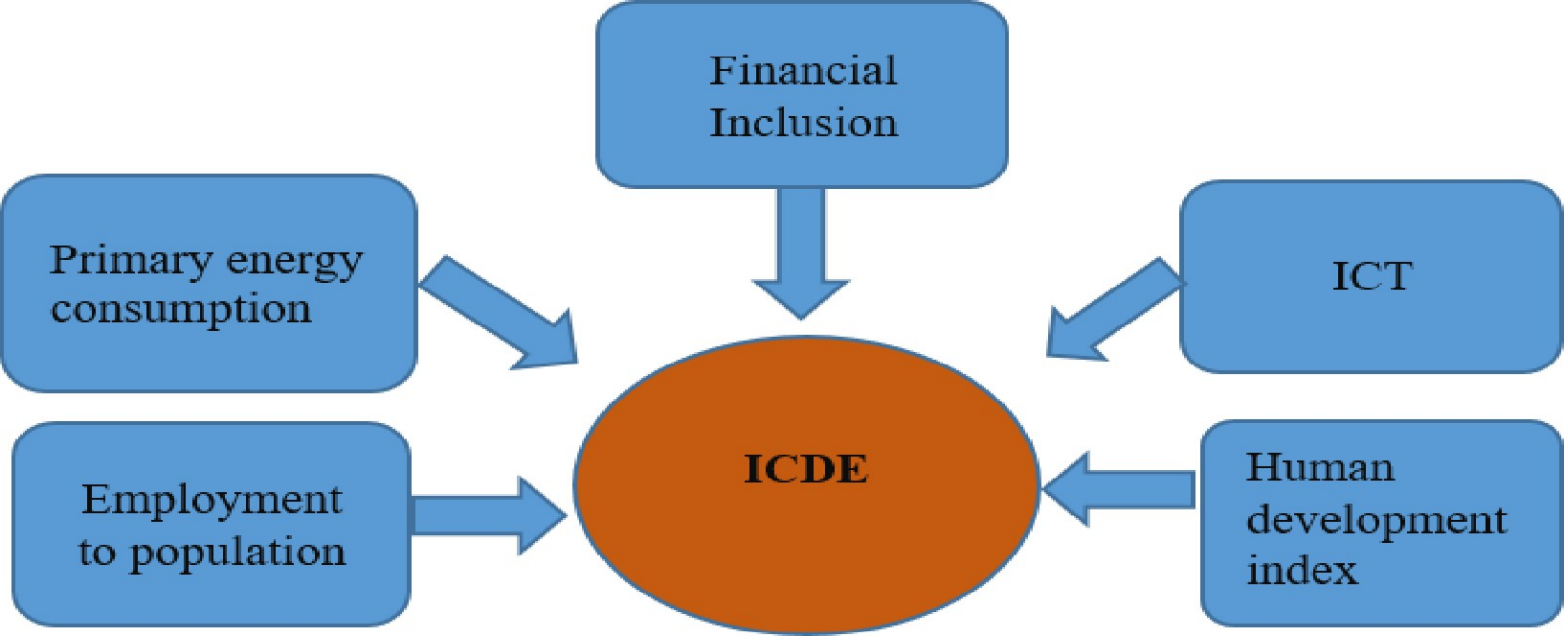
Theoretical framework.

It focuses on the theoretical underpinnings of financial inclusion’s role in facilitating economic development. Understanding economic progress helps to reduce poverty by promoting an inclusive financial system [54]. Indeed, financial inclusion contributes significantly to economic growth by assuring optimal resource allocation, pooling and mobilization of savings, and improved investment estimation. A well-developed financial inclusion system expands savings and investment options, contributing to economic progress. However, it is crucial to recognize that financial inclusion poses environmental challenges, prompting a discussion of long-term development goals. Conversely, advances in the finance sector make technical improvements more accessible, which can cut pollutant carbon emissions by encouraging the effective consumption of energy equipment. This suggests that energy efficient products and technology improvements can help address environmental degradation. Financial inclusion can help with sustainable development efforts by increasing the adoption of such technology [55]. Financial inclusion is especially important for disadvantaged groups such as farmers and low-income families. Access to cheap financial goods and services can substantially affect these populations’ livelihoods and general well-being. Regarding environmental sustainability, financial inclusion can encourage these vulnerable people to invest in cutting-edge technology to prevent pollution [56]. However, it is critical to recognize that financial inclusion can damage the environment. One of the potential negatives is an increase in the consumption of energy-intensive commodities, which could result in increased emissions and environmental degradation. Individuals and groups may be able to acquire and use more energy-intensive products, such as autos, appliances, or other things that contribute to increased carbon emissions when they get financial services access. Furthermore, greater financial inclusion can incentivize businesses to increase output to meet rising demand. This can potentially increase pollutant emissions and enhance the negative environmental effects of industrial activities. Fig 1, represents the theoretical framework of study.

Theoretical research has highlighted three primary ways information and communication technology can affect the environment [57]. 1) The environmental effects of ICT device manufacture, consumption, and reutilizing are direct impacts. The extraction of raw materials and energy usage during the production process of ICT equipment can have major environmental consequences. 2) Indirect effects are caused by the environmental repercussions of employing ICT in numerous industries, such as manufacturing, transportation, and construction. Through technical breakthroughs and better energy efficiency, ICT can help to reduce pollution emissions. 3) Tertiary effects are the long-term implications of ICT adoption, which might alter economic structures and consumer patterns. The increasing use of ICT can cause changes in industries, labour markets, and lifestyles. Regarding tertiary impacts, adopting ICT might result in behavioural changes due to increased efficiency and lower prices of ICT products. This can lead to increased consumption, including the use of energy-intensive products. As a result, there is a chance that pollutant emissions will rise. As a result, when establishing environmental policies to reduce pollutant emissions from the ICT sector, it is critical to consider consumption patterns [20]. Numerous studies have found evidence that ICT and financial inclusion positively influence economic growth. [58], among other studies, have discovered empirical support for this association. The spread of ICT is critical in integrating the impact of financial inclusion on economic success. This integration occurs through decreasing information and transaction costs, making financial services more accessible to individuals and enterprises. Furthermore, ICT infrastructure allows for effective corporate control, a critical role of financial intermediaries. However, just a few research has looked into whether the spread of ICT strengthens the impact of financial innovation on environmental damage. In a study by [41], It is proposed that technology innovation in the ICT industry can mitigate the negative environmental consequences of financial innovation. This demonstrates the potential of ICT innovation to provide underprivileged individuals with affordable financial solutions while implementing technological advances to reduce pollutant emissions. The EKC framework has been utilised to investigate the relationship between financial inclusion and environmental degradation. The EKC hypothesis, proposed by [48], suggests that environmental degradation increases in the initial stages of economic development but reduces when economic success is made, allowing resources to be focused on environmental protection. Previous research has looked into the EKC assumption validity by relating environmental degradation to GDP as a measure of progress. However, it is vital to remember that human life quality is affected by real per capita GDP increases and aspects such as healthcare and education [59, 60]. As a result, the human development index, which incorporates various development dimensions such as education and health, is seen as a more accurate indicator of development [59]. Energy is essential to economic activity and advancement but has negative environmental consequences. Increased energy consumption due to economic growth may result in higher pollutant emissions. much research has found an association between energy usage and environmental damage [52, 60]. The increased consumption of energy caused by urbanization adds to environmental depletion. Furthermore, widespread agreement exists that population expansion contributes greatly to environmental degradation [61]. Population growth increases resource consumption, energy demand, and pollution levels, negatively influencing the environment. This study investigates the significance of financial inclusion in reducing pollution emissions in oil-producing countries. The analysis is based on the EKC phenomenon’s theoretical framework. Eq 1 is the model specification for this study:

ICDE=F(HDI,EMPO,PECN,ICT,INFI) (1)

Several variables are taken into account in the model specification. The human development index [62] is provided as a measurement, captures the potential nonlinear relationship between economic development and environmental degradation. The employment-to-population ratio (EMPO) is a population proxy. PECN is a proxy for primary energy consumption. ICT is information and communication technology index measured by mobile cellular subscriptions and internet penetration. Financial inclusion (FI) is the index of two indexes: the financial institution’s index (FII) and the financial institution’s depth index (FIDI). The carbon intensity indicator (ICDE) shows the intensity of carbon emissions as measured by CO_2_/GDP using 2015 exchange rates (kg CO_2_/USD). The logarithmic forms of each variable are supplied to ensure the analysis’s robustness. This transformation is useful for dealing with data with a large range of values and capturing potential nonlinear interactions between variables. Eq 2 represents the altered model.


$${LNICDE}_{it}=a_{i}+{\beta_{1}LNHDI}_{it}+\beta_{2}\;{LNEMPO}_{it}+\beta_{3}{LNPECN}_{it}+\beta_{4}{LNICT}_{it}+\beta_{5}{LNINFID}_{it}+\varepsilon_{it}.$$ (2)

I denote the countries, where t defines the time; LN signifies the natural logarithm; I denote the variables intercept; it signifies the error term; and s’ are examples of regression Coefficient to be determined [63]. According to [64], the Coefficient of energy consumption and population are both likely to be positive. (3 > 0, 4 > 0). Regarding its impact on carbon intensity, the ICT diffusion Coefficient sign might be negative or positive [27], i.e., β4 > 0 or < 0. Financial inclusion can contribute to or mitigate adverse environmental effects [20] β5 > 0 or < 0. Among the several ICT indicators examined, Internet- and mobile use expansion is rising in the nations studied.

### 3.2 Panel data estimation technique

This study used a set of methodologies to analyse the impact of the HDI, energy consumption, population, financial inclusion, and ICT spread on environmental degradation. The main steps were: Check for CSD: We started by looking for cross-sectional dependencies between these variables. It is critical to address this issue since disregarding cross-sectional dependence can result in biased or false conclusions. We utilized the cross-sectional dependency (CSD) test to recognize potential CSD [65]. This test can assist in determining whether a cross-sectional dependency problem exists. We used second-generation unit root testing in the second stage to generate robust estimates. These tests allow us to assess the variables’ stationarity and verify the results’ dependability. We used FMOLS, FE-OLS, and quantile regression (QR) methods for panel data with fixed effects to examine the heterogeneous covariance conditional influence of the predictors on carbon intensity. This method allows us to consider individual-specific effects and conditional heterogeneity across quantiles. The test statistics for cross-sectional dependence were calculated using the text’s specific equation (Eq 3). Regrettably, the equation itself is not supplied in the context.


$$CD=\frac{\sqrt{2T}}{N(N-1)}\sum_{i=1}^{N-1}\sum_{j=i+1}^{N}{\hat{\rho }}_{ij}$$ (3)

Where ${\hat{\rho }}_{ij}$ represents the correlation of residuals

A preliminary test to know the variables’ static qualities is also necessary. The second generation panel unit root tests are required to achieve valid Co-efficient estimates when cross-sectional dependence exists. This study utilized the cross-sectional augmented unit root test proposed to address the effects of heterogeneity and CSD to avoid biased results. [66], known as the CIPS test. The assessment of the CIPS unit root test, as described by [65], is represented by the following equation (Eq 4):

$${\Delta Y}_{it}={\Delta \emptyset }_{it}+\beta_{i}X_{it-1}+\vartheta_{i}T\sum_{j=1}^{n}\theta_{ij}{\Delta X}_{it-j},\varepsilon_{it}$$ (4)

∅ represents the intercept at time t for each cross-sectional unit I. At time t, *X*_*it*_ denotes the interest of each variable for the cross-sectional unit I at time t Δ: Denotes the difference operator, which computes a variable’s initial difference. T, is the period of interest and represents the number of periods in the panel. *ε*_*it*_ At time t, it represents the error term for each cross-sectional unit I. The equation calculates the CIPS unit root test, which accounts for cross-sectional dependence in panel data and evaluates the presence of unit roots in variables. If β1 > 0 and β2 > 0, the presence of the EKC phenomena is confirmed by the statement in Eq 2. Traditional pooled techniques or pooled ordinary least squares improved with Kraay and Driscoll standard errors, on the other hand, have drawbacks, as noted by [67]. To circumvent these constraints, the QR with fixed effects described is used in this study [68]; this yields more accurate findings. This method is beneficial when there is a weak or non-presence relationship between the variables conditional means under consideration. Furthermore, non-crossing evaluations of the regression quantiles are provided by quantile regressions. As a result, the QR technique enables us to discover the conditional heterogeneous covariance impacts of carbon emissions indicators by considering individual effects that affect the entire distribution rather than simply the moving averages. In this investigation, the fixed effects QR equation, as stated in Eq 5, is used:

$${QY}_{i,t}{(\tau |X}_{it}=\alpha {(\tau )\prime X}_{it}+\beta_{i},\;i=1,\ldots \ldots ,N,t=1,\ldots ..,T$$ (5)

For Eq 5, *Y*_*it*_ represents the predicted variable (LNICDE), *X*_*it*_ denotes the regresses, a (τ) represents the unknown coefficients, and *β*_*i*_ Refers to the personal effects. The authors developed the models presented below to examine the impact of the HDI, EMPO, PECN, financial and ICT diffusion, and inclusion on carbon intensity:

### 3.3 Slope homogeneity tests

When conducting panel data analysis, which combines cross-sectional and time-series elements, it is essential to consider possible causes of variation, such as individual-specific influences. Disregarding such diversity can indeed result in prejudiced outcomes. Pesaran and Yamagata [69], introduced a test for slope homogeneity in panel data with a large number of cross-sectional units (N) and time periods (T), using a standardized version of Swamy’s test. The test assumes that εi,t and εj,s are independently distributed when i is not equal to j and/or t is not equal to s, but it allows for a variance that varies across different cases. The test statistics is given by

$$\hat{\Delta }=\frac{1}{\sqrt{N}}(\frac{\sum_{i=1}^{N}\tilde{d_{i}}-k_{2}}{\sqrt{2k_{2}}})$$ (6)

Eq (6) defines $\tilde{d_{i}}$ as the weighted discrepancy between the cross-sectional unit specific estimate. For error terms that follow a normal distribution, the adjustment for bias in the mean variance $\tilde{\Delta }$ can be mathematically represented as:

$$\hat{\Delta }=\sqrt{N}(\frac{N^{-1}\sum_{i=1}^{N}\tilde{d_{i}}-k_{2}}{\sqrt{Var(\tilde{Z_{i}}}T_{i}})$$

Where

$$Var(\tilde{Z_{i}}\;T_{i})=\frac{2\;K_{2}(T_{i}-k-1)}{T_{i}-k_{1}-1}$$

### 3.4 Data collection

**Fig 2:**
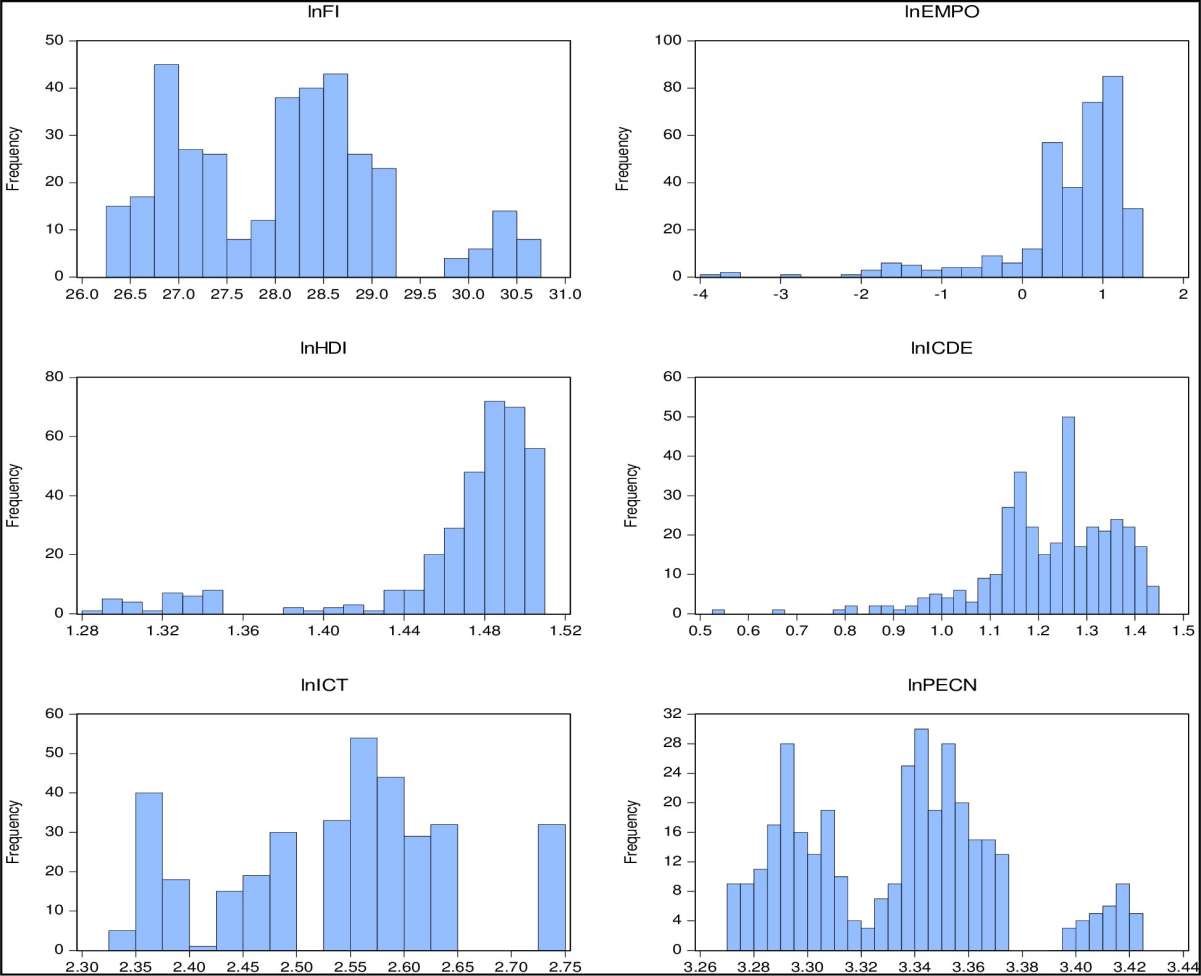
Frequency distribution.

Using annual balanced panel data from the ten top oil exporting countries: the UAE, Kazakhstan, Russia, Saudi Arabia, Norway, Kuwait, Canada, Nigeria, Iraq, and the USA. We investigate the impact of the HDI, energy consumptions, population, financial inclusion which is the index of FII and FIDI, and ICT diffusion which is the index of MPH and IUI, on environmental degradation. These countries were picked because of their oil reserves, lower crude oil prices, and higher revenue to import more energy-intensive items. Based on data availability, the data spans the years 1990 through 2021. The chosen time-period and the selection of oil-producing countries for this study are motivated by the recognition of a critical juncture in global economic and technological evolution, coupled with the unique challenges posed by the economic reliance on oil. The UN Development Programme provided the annual HDI data, while the World Bank provided information on the employment-to-population ratio, Internet users, and mobile cellular subscribers [70]. Financial inclusion data were retrieved from the International Monitoring fund, and energy consumption data were collected from British Petroleum [71]. Fig 2, represents the frequency distribution of the variables. The International Energy Agency provided the data for the indicator of carbon intensity (ICDE) [72]. Table 1 provides detailed information on the variables used in the study, and Table 1 presents the variable definition and sources.

**Table 1:** Variable used in the study.

Variables	Definition	Sources
LNIUI	${Individual\;using\;Internet\;\%\;of\;(population)}_{.}$	WB
LNMPH	${Mobile\;cellular\;subscription\;(per\;100\;people)}_{.}$	WB
LNEMPO	${Employment\;to\;population\;ratio,\;15,\;\%\;of\;total\;(model\;ILO)}_{.}$	WB
LNFII	${Financial\;Institutions\;Index}_{.}$	IMF
LNFIDI	${\;Financial\;Institutions\;Depth\;Index}_{.}$	IMF
LNPECN	${Primary\;energy\;Consumptions\;(-tons\;of\;oil\;equivalent)}_{.}$	BP
LNHDI	${Human\;development\;index}_{.}$	UNDP
LNICDE	${CO2/GDP\;using\;exchange\;rates\;kg\;CO2/USD}_{.}$	IEA

## 4. Empirical results and discussions

### 4.1 Descriptive statistics

Table 2 provides descriptive statistics for the variables of interest. The mean values for HDI, FI, EMPO, ICDE, ICT, and PECN are 0.099, 0.507, 1.770, 0.284, 0.670, and 0.667, respectively. Additionally, the maximum values observed for these variables are 2.841, 2.219, 2.289, 0.708, 2.007, and 1.989, respectively. A noteworthy finding from Table 2 is the p-values obtained from the Jarque–Bera test, all of which are greater than 0.05. This indicates a rejection of the assumption of abnormality in the data. As a result, relying on conventional estimation techniques in this scenario may lead to potentially misleading outcomes.

**Table 2:** Descriptive statistics.

Var	LNHDI	LNFI	LNEMPO	LNICDE	LNICT	LNPECN
Mean	0.099	0.507	1.770	0.284	0.670	0.667
St. d	0.185	0.600	0.089	0.377	1.043	0.639
Max	2.841	2.219	2.289	0.708	2.007	1.989
Min	0.386	-2.666	1.551	-1.000	-4.706	-0.780
Skew	1.235	-0.038	0.188	-0.112	-0.797	0.690
Kurt	1.998	3.112	3.025	2.949	2.373	2.455
Jarq-Bera	71.354	51.991	97.846	34.145	58.842	29.160

LNHDI, LNFI, LNEMPO, LNICDE, LNICT, and LNPECN represent. The average value of the natural logarithm of the Human Development Index, Financial Inclusion index, Employment Rate, CO_2_ emission, Information and Communication Technology. Percentage of Energy from Clean Sources.

Fig 1 complements these findings by visually representing the data, confirming the non-normality as indicated by the J-B test results. Given this non-normality, using linear estimation techniques may not yield reliable results. Therefore, it is crucial to consider employing quantile regression, which is better equipped to address issues related to different quantiles of the data distribution

### 4.2 CD and unit root test results

The evaluation of the data begins with diagnostic testing. The first test is performed to look for CSD in the variables, using the technique developed by [65]. The initial CSD test, which evaluates the interconnectedness between the selected nations, is summarised in Table 3. Table 3 demonstrates the rejection of null hypothesis of no CSD, employing that variables are under consideration of CD exhibit. This means that the oil-exporting countries studied are linked via multiple economic pathways. It implies that a shock in one country can influence the other nine. In other words, previously unrecognized shared financial inclusion (FI) variables and ICT diffusions in one selected country are transferred to others. CSD is ubiquitous in longitudinal data and, if not handled, can dramatically limit panel data processing efficiency and result in biased conclusions. Given the cross-sectional in all variables, the study utilized second-generation unit root tests to get reliable estimations. The study applies the cross-sectional augmented (CIPS) panel unit root test established to analyse the integrating features of the variables more precisely [66]. The outcomes of the second-generation panel unit-root test for the panel variables are shown in Table 3.

**Table 3:** Cross-sectional dependence results.

Var	Pesaran CD	B-P LM	Prob	Hypothesis
LNHDI	26.46a	1004.6a	0	The H0 hypothesis is rejected
LNFI	17.00a	460.53a	0	The H0 hypothesis is rejected
LNEMPO	51.55a	553.75a	0	The H0 hypothesis is rejected
LNICDE	27.69a	435.54a	0	The H0 hypothesis is rejected
LNICT	44.48a	656.289a	0	The H0 hypothesis is rejected
LNPECN	17.75a	611.66a	0	The H0 hypothesis is rejected

Note: a p-value < 0.01., b p-value < 0.05, and c, p-value < 0.10, LN-natural logarithm

The HDI, PECN, and ICDE all are non-stationary at their current levels, according to Table 4. The null hypothesis of a unit root could not be rejected for these variables in their original form. However, when the initial difference is included, the CIPS test, which accounts for CSD, reveals that all variables become stationary. Consequently, we may reject the H0 of the unit root for all variables in their first difference I (1). As a result, the variables employed in this study are first-difference I (1) stationary and have a similar integration order.

**Table 4:** Unit root test.

Var	Level	1st Diff	Levels	1st Diff
	No trend	With trend	No trend	With trend
LNHDI	-1.98	-1.72	-3.10a	-2.86b
LNEMPO	-2.57a	-2.33	-3.78a	-2.08b
LNPECN	-1.39	-2.97	-5.21a	-5.47a
LNICT	-2.48a	-2.64a	-3.11a	-3.27a
LNFI	-3.21a	-2.45a	-3.49a	-3.87a
LNICDE	-1.99	-2.55	-4.66a	-5.36a

Note: a = p < 0.01, b = p < 0.05, c = p < 0.10.

The hypothesis of coefficient heterogeneity, or the variation in coefficients, is examined by the "Delta Test" as an alternative to the Swamy test for slope homogeneity. The calculation of the Delta test statistic and the adjusted bias version of the mean and standard deviation of Delta statistics is shown in Table 5:

**Table 5:** Slope of homogeneity.

Test	Statistics	p-value
Homogeneity Test (H0: slope coefficients are homogenous)		
Δ	-0.175	0.861
Δ_adj_	-0.203	0.835

The null hypothesis, which posits homogeneity of slopes, was evaluated against the alternative hypothesis of heterogeneity. The results of the test [-0.175 (0.861), -0.203 (0.835)] indicate that we reject the null hypothesis at a 0.05 level of significance. This implies that there is significant evidence in favor of individual-specific effects influencing the relationship between variables. Consequently, it is appropriate to conclude that the assumption of homogeneity of slopes is not tenable in this context. As a result, the model incorporating fixed/random effects is deemed more appropriate for capturing the nuances of individual-specific influences on the relationship under investigation. This finding underscores the importance of accounting for non-observed heterogeneity in subsequent panel data analyses to ensure unbiased and robust results.

### 4.3 Panel co integration analysis

Co Integration tests are utilized in econometrics to evaluate the enduring correlation between two or more non-stationary time series variables [24]. When evaluated separately, non-stationary variables may display trends or random walks, which makes it difficult to determine their correlations. Co Integration aids in determining if a linear combination of these variables produces a stationary series, indicating a stable, long-term relationship. To assess the robustness of the findings, sensitivity analyses were performed, considering different specifications and parameterizations. The choice of lag length, the inclusion of additional control variables, and other model specifications were systematically evaluated. The analysis culminated in a comprehensive interpretation of the Pedroni co integration results, shedding light on the existence and nature of co integration relationships among the economic variables under investigation in the panel dataset. The methodological approach adopted in this study aimed to provide a thorough and reliable exploration of long-term dynamics within the panel context. A statistically significant and positive trace statistic, along with the associated eigenvalue statistics, would suggest the presence of cointegration [73]. This implies that there exists a stable long-term relationship among the variables, supporting the notion of a shared equilibrium [74]. Coefficients associated with the co integrating vectors provide information about the strength and nature of the long-term associations. Positive coefficients indicate a positive relationship, while negative coefficients suggest an inverse relationship. The magnitude of these coefficients reflects the extent to which changes in one variable affect the others in the long run. Table 6 shows the results of long-run co-integrations.

**Table 6:** Long-run co-integration test findings co integration all tests are significant.

	t-stat	P-value
**Modified Phillips-Perron**	2.2664	0.0117
**Phillips-Perron**	-3.1343	0.0009
**ADF**	-1.7452	0.0405

### 4.4 Quantile regression results

**Fig 3:**
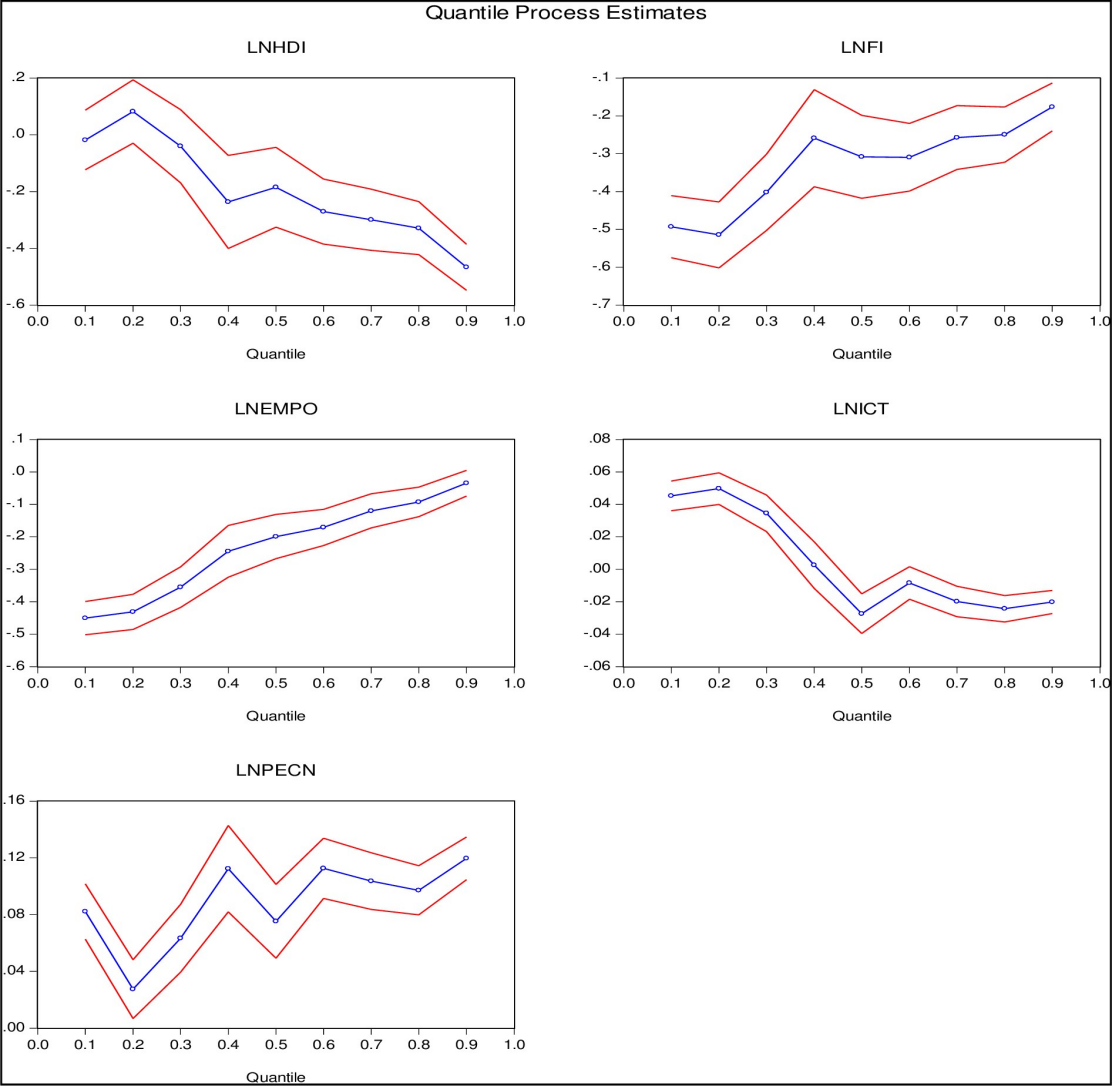
Plotting outcomes from panel quantile regression.

Table 7 presents the findings of panel quantile estimation, which analyses the relationship between various factors and ICDE across different quantiles (1st to 9th). The study’s first important outcome indicates that economic growth, as measured by the human indexes, considerably influences carbon intensity (ICDE) through all quantiles. The findings support the Environmental Kuznets Curve (EKC) theory for all nations and show an inverted U-shaped link between economic growth and environmental degradation (suggested citations) [23, 27]. The degradation of the environment and income is on the rise in the early phases of economic development [75]. However, after a positive economic growth level is reached, income is channelled toward environmental protections, decreasing environmental degradation [76, 77]. These findings provide credence to the concept that economic progress fosters environmental sustainability. Regarding the human development index Co-efficient, the result demonstrate a significant and positive relationship with carbon intensity across all quantiles, with a declining movement [78, 79]. The second finding are negative and statistically significant link between financial inclusion and ICDE. The outcomes suggest that increasing financial inclusion can lower carbon intensity in the long run. Financial inclusion statistically influences ICDE at all levels, with a diminishing pattern observed This implies that higher levels of financial inclusion have a larger potential to reduce carbon intensity, particularly in low-carbon countries [24]. Financial inclusion’s negative coefficient value can be linked to increased energy efficiency, technological development access, and the start-up of renewable energy innovation initiatives made possible by financial inclusion. These findings align with [20, 27], they find a negative link between financial inclusion and ICDE in quantiles higher than the first. The third finding demonstrate a negative relationship between employment to population ratio and carbon intensity, decreasing Co-efficient over quantiles. These findings imply that energy consumption increases when economic advancement grows with a larger employment-to-population ratio, resulting in higher carbon emissions [78–80]. The Previous research of [79] has found that the impact of population on environmental degradation is consistent. At the same time, the opposite trend implies that as we move from lower to higher quantiles, the effect of the EMPO ratio on ICDE declines. The study’s fourth finding examines ICT’s direct effects on ICDE. The QR results show a negative, statistically significant association between ICT usage and carbon intensity across all quantiles. For example, increased mobile cellular subscribers are connected with decreased carbon intensity. In contrast, increased Internet users (as a percentage of the population) are likewise associated with decreased carbon intensity. The findings support the indirect influence of ICT use on carbon intensity, which includes enhancing energy efficiency, advancing technology, and contributing to renewable energy innovation projects. These findings align with earlier research, such as [20], who discovered that using the internet greatly reduced pollution emissions. The study’s last major conclusion demonstrates that PECN statistically significantly and favourably influences ICDE across all quantiles in models 1–4. PECN coefficients show a declining trend across quantiles, showing that the impact of PECN on ICDE decreases as we movement from lower to higher quantiles. This research emphasizes the environmental stress caused by these countries’ energy usage. This result agrees with the findings of [80], who discovered an association between non-renewable energy consumption and economic performance but also discovered a negative environmental impact on OECD countries. Similarly, The significance of reducing fossil fuel-based energy usage to reduce greenhouse gas emissions was emphasized [81]. Fig 3, exhibits the Plotting outcomes from panel quantile regression

**Table 7:** Panel quantile regression model results.

Variables	Values	Grids of Quantile								
		0.1	0.2	0.3	0.4	0.5	0.6	0.7	0.8	0.9
**LNHDI**	**Co-eff**	0.043^a^	1.116^a^	1.288^a^	1.394^a^	1.460^a^	1.548^a^	1.770^b^	1.654^c^	1.148^c^
	**Std. Er**	0.015	0.430	0.412	0.359	0.352	0.380	0.828	5.182	4.050
	**t-Stat**	2.896	2.594	3.129	3.879	4.145	4.075	2.137	0.319	0.283
** **	**Prob.**	0.004	0.010	0.002	0.000	0.000	0.000	0.033	0.050	0.054
**LNFI**	**Co-eff**	-0.32^a^	-0.94^a^	-0.90^a^	-0.73^a^	-0.65^a^	-0.62^a^	-0.73^a^	-0.77^b^	-0.76^b^
	**Std. Er**	0.054	0.151	0.117	0.104	0.093	0.085	0.158	0.631	0.518
	**t-Stat**	-5.935	-6.236	-7.705	-7.046	-7.103	-7.243	-4.650	-1.230	-1.478
** **	**Prob.**	0.000	0.000	0.000	0.000	0.000	0.000	0.000	0.020	0.040
**LNEMPO**	**Co-eff**	-0.65^a^	-0.71^a^	-0.56^a^	-0.38^a^	-0.27^a^	-0.19^a^	-0.17^b^	-0.14^c^	-0.17^c^
	**Std. Er**	0.034	0.039	0.095	0.086	0.057	0.050	0.082	0.321	0.243
	**t-Stat**	-18.99	-18.18	-5.924	-4.523	-4.755	-3.801	-2.116	-0.445	-0.703
** **	**Prob.**	0.000	0.000	0.000	0.000	0.000	0.000	0.035	0.057	0.083
**ICT**	**Co-eff**	-0.020	-0.013	-0.017	-0.045	-0.07^a^	-0.09^a^	-0.08^a^	-0.08^a^	-0.06^b^
	**Std. Er**	0.024	0.018	0.029	0.029	0.022	0.022	0.052	0.069	0.044
	**t-Stat**	-0.848	-0.716	-0.597	-1.562	-3.253	-4.113	-1.639	-1.256	-1.493
** **	**Prob.**	0.397	0.475	0.551	0.119	0.001	0.000	0.012	0.010	0.037
**LNPECN**	**Co-eff**	0.331^a^	0.38^a^	0.288^a^	0.23^a^	0.15^a^	0.13^a^	0.15^a^	0.12^a^	0.09^a^
	**Std. Er**	0.030	0.039	0.057	0.055	0.039	0.037	0.045	0.036	0.028
	**t-Stat**	11.110	9.939	5.048	4.134	3.978	3.528	3.412	3.200	3.326
** **	**Prob.**	0.000	0.000	0.000	0.000	0.000	0.001	0.001	0.002	0.001

Note: a = (p < 0.01), b = (p < 0.05), c = (p < 0.10), and QR, quantile regression

## 5. Robustness analysis

The robustness analysis conducted in this study aimed to assess the stability and consistency of the key findings by subjecting the model to various sensitivity tests and alternative specifications. In our robustness analysis, we utilized the Financial Inclusion Index (FII) as the dependent variable. The Financial Inclusion Index was developed by combining two variables, namely the Financial Institutions Index and the Financial Institutions Depth Index, using principal component analysis. The objective was to evaluate the robustness of the results under different conditions, ensuring that the conclusions drawn from the primary model were not unduly influenced by specific assumptions or model choices. Across the spectrum of robustness checks, the central findings held firm, thereby enhancing the confidence in the validity and reliability of the results.

### 5.1 Hausman test

The Hausman test is used as a crucial validation step for the selected econometric model, specifically to evaluate whether the fixed effects or random effects specification is more appropriate. The test is crucial in assessing the validity of the assumptions that underlie these specifications, hence impacting the dependability of the computed coefficients. The null hypothesis, which assumes that the preferred model is random effects, is compared to the alternative hypothesis that the fixed effects model is better suitable. The decision to either reject or fail to reject the null hypothesis yields valuable insights into the possible existence of unobserved differences among individuals.

According to the findings (chi sq 2 (4) 180.66), prob. Chi 2 (0.000) the null hypothesis is rejected in favor of the fixed effects model, it indicates that unobservable characteristics that are unique to each entity have a significant impact on the dependent variable. It is crucial to consider entity-specific effects in order to provide unbiased and efficient parameter estimations. Table 8 shows the results of regression model estimations.

**Table 8:** Results of regression model estimations.

Regression Model Estimations				
Dependent Variable is Financial Inclusion Index (FII)				
	I		II	
Regressors	Fixed Effect Estimations (Within regression)		Fixed Effects Estimation with Driscoll and Kraay Standard Errors	
	Coefficient	p-value	Coefficient	p-value
**lnICT**	-0.032359 (0.210712)	0.126	-0.0323259 (0.016590)	0.60
**lnHDI**	-0.009665 (0.055179)	1.861	-0.0096656 (0.014343)	-0.505
**lnPECN**	-.48541 (0.067548)	0.000	-0.48541 (0.101427)	0.000
**lnEMPO**	2.696951 (0.258298)	0.000	2.696951 (0.495192)	0.000
**C**	-10.77873 (1.037019)	0.000	-10.77873 (2.047615)	0.000
	R^2^	0.304	R^2^	0.3045
	F(4, 306)	33.49	Wald χ2 (4, 31)	20.86
	p-val > F	0.000	p-val > χ2	0.000

Notes: Parentheses Contain Standard Error

In the fixed effects model estimates within regression, the variables PECN (0.000) and EMPO (0.000) are statistically significant at a 1% level of significance, indicating a strong relationship. However, the variables ICT (0.126) and HDI (1.861) are not statistically significant, suggesting that they do not have a substantial impact on the outcome. Nevertheless, the estimates obtained from the fixed effect model using Driscoll and Kraay standard error exhibit a similarity to the results obtained from the regression. These findings are consistent with established theoretical and empirical literature [76]. The coefficient of determination, R^2^, for within regression and with Driscoll and Kraay Standard Errors is 0.3045, indicating that 30% of the variability in the dependent variable can be accounted for by the explanatory variables, while the rest variability is attributed to variables that were not included in the model.

## 6. Conclusion and policy suggestions

From 1990 through 2021, this study looked at the relationship between financial inclusion, ICT diffusion, economic growth, and environmental deterioration in a panel dataset of ten oil-producing countries. The findings revealed that these countries, well known for their major contribution to environmental deterioration, must balance economic expansion and environmental preservation. ICT has been suggested as a potential instrument for mediating this relationship and improving environmental initiatives in these countries. For all oil-producing countries, the analysis proved the existence of an inverted U-shaped Environmental Kuznets Curve (EKC). This implies that beyond a certain degree of development, countries prioritize environmental concerns over further economic progress. The study also found that varying quantiles of energy usage have a negative influence on the environment. The importance of ICT and financial inclusion in enhancing environmental quality has been emphasized. The interplay between ICT and financial inclusion positively impacted the environment. As a result, it is suggested that these countries use their natural resources to support economic growth while avoiding environmental deterioration. These countries are suggested to improve their renewable energy consumption by promoting activities that use renewable energy resources to address the energy consumption patterns that lead to environmental degradation.

Policymakers should strike a balance between financial inclusion and ecological preservation requires regulations that incentivize environmentally responsible financial practices and discourage activities that contribute to environmental degradation. Financial inclusion activities that are high technology innovations and environmentally friendly can be critical in reaching this goal. Governments and politicians are encouraged to create policies that promote energy management and reduction, financial inclusion, and ICT utilization. Public awareness efforts should educate people about the negative environmental implications of energy usage while emphasizing the benefits of financial inclusion and ICT. Policymakers are encouraged to aggressively advocate for the implementation of sustainable technologies in the financial industry. Allocating funds towards environmentally sustainable digital infrastructure, alongside the advancement of digital payment systems that have minimal negative effects on the environment, might help reduce the harmful environmental consequences linked to financial operations. Policymakers should engage in cooperation with technology providers to guarantee the advancement and implementation of ICT solutions that are in line with environmental sustainability objectives. While this work adds to the empirical and theoretical literature, there are certain limitations that future research could address. Finally, it is essential to incorporate environmental impact evaluations into the regulatory approval procedure for financial and technological initiatives. This guarantees that possible environmental repercussions are methodically assessed and taken into account during the decision-making process. Policymakers ought to engage in cooperation with environmental specialists and business stakeholders in order to develop all-encompassing evaluation frameworks that direct the authorization of financial and technological initiatives in nations that produce oil. Future research should include importing countries in the analysis, capturing multiple effects connected to ICT, financial inclusion, and the environment. Furthermore, full financial inclusion measures like depth and access would improve understanding of its impact.

## Supporting information

S1 File (DOCX)
