# Expression of Concern: Does financial inclusion and information communication technology affect environmental degradation in oil-producing countries?

**DOI:** 10.1371/journal.pone.0323862

**Published:** 2025-05-01

**Authors:** The PLOS One Editors

The *PLOS One* Editors issue this Expression of Concern to inform readers that we have concerns about this article’s [1] peer review, and about conclusion statements referring to causative (as opposed to correlative) relationships that overreach what is supported by the reported research.

In addition, please note that Reference 35 [2] was retracted after this article [1] was published.
