# Supplementary material for: Does financial inclusion and information communication technology affect environmental degradation in oil-producing countries?
Source: PLoS One. 2024 Mar 20;19(3):e0298545. doi: 10.1371/journal.pone.0298545 (PMC10954129; doi:10.1371/journal.pone.0298545)
Supplement: S1 File — (DOCX) [file pone.0298545.s001.docx]

**Data Information**

| **Variables** | **Definition** | **Sources** |
| --- | --- | --- |
| LNIUI | $Individuals$ | WB <https://data.worldbank.org/>; |
| LNMPH | ${Mobile cellular subscription (per 100 people)}_{.}$ | WB<https://data.worldbank.org/>; |
| LNEMPO | ${Employment to population ratio, 15, \% of total (model ILO)}_{.}$ | WB<https://data.worldbank.org/>; |
| LNFII | ${Financial Institutions Index}_{.}$ | IMF<https://www.imf.org/en/Data> |
| LNFIDI | ${Financial Institutions Depth Index}_{.}$ | IMF<https://www.imf.org/en/Data> |
| LNPECN | ${Primary energy \mathrm{Consumption} (-tons of oil equivalent)}_{.}$ | BP <https://www.bp.com/> |
| LNHDI | ${Human development index}_{.}$ | UNDP<https://www.undp.org/> |
| LNICDE | ${\mathrm{CO}2/GDP using exchange rates kg CO2/USD}_{.}$ | IEA<https://www.iea.org/data-and-statistics> |
